# Artificial Intelligence as a Diagnostic Tool in Preoperative Surgical Planning for Early Non-Small Cell Lung Cancer: A Single-Center Experience

**DOI:** 10.3390/jcm14217609

**Published:** 2025-10-27

**Authors:** Zeljko Garabinovic, Milan Savic, Nikola Colic, Jelena Rakocevic, Maja Ercegovac, Milos Mitrovic, Katarina Lukic, Jelica Vukmirovic, Jelena Vasic Madzarevic, Stefan Stevanovic, Gordana Bisevac Peric, Miljana Bubanja, Aleksandra Pavic

**Affiliations:** 1 Clinic for Thoracic Surgery, University Clinical Center of Serbia, 11000 Belgrade, Serbia; 2 Medical Faculty, University of Belgrade, 11000 Belgrade, Serbia; 3 Center for Radiology and MR, University Clinical Center of Serbia, 11000 Belgrade, Serbia; 4 Institute of Histology and Embryology “Aleksandar Đ. Kostić”, Faculty of Medicine, University of Belgrade, 11000 Belgrade, Serbia; 5 Clinic for Thoracic Surgery, University Clinical Center of Niš, 18000 Niš, Serbia; 6 Clinic for Pulmonology, University Clinical Center of Serbia, 11000 Belgrade, Serbia

**Keywords:** artificial intelligence, early NSCLC, surgery

## Abstract

**Background**: Lung cancer remains the leading cause of cancer-related mortality worldwide, with non-small cell lung cancer (NSCLC) accounting for the majority of cases. Radiomics and artificial intelligence (AI) have emerged as promising tools for quantitative imaging analysis and precision staging. This study aimed to evaluate the ability of an AI-based radiomics model to preoperatively predict tumor (T) and nodal (N) stage, lymphovascular invasion (LVI), and postoperative complications in patients with early-stage NSCLC. **Material and Methods**: This retrospective study included 51 consecutive patients who underwent anatomical lobectomy with systematic lymph node dissection between 2019 and 2024, at the Clinic for Thoracic Surgery of the University Clinical Center of Serbia. Quantitative imaging features were extracted from preoperative CT scans using the Lesion Scout with Auto ID module (syngo.via VB50 MM, Siemens Healthineers). Radiomics and clinical predictors were analyzed using regularized logistic regression (LASSO) with five-fold cross-validation. Model performance was assessed using AUC, accuracy, sensitivity, specificity, precision, and F1 score, and calibration was evaluated using the Hosmer–Lemeshow test. Groups were compared using parametric and non-parametric tests. Correlation between the variables was assessed using Spearman’s rank correlation coefficient. All *p*-values less than 0.05 were considered significant. **Results**: The AI-based model showed excellent performance for predicting the T component (training AUC = 0.89; test AUC = 0.86; F1 = 0.81) and acceptable calibration (*p* = 0.41). Nodal metastasis (OR = 0.108; 95% CI: 0.011–1.069; *p* = 0.057) and LVI (OR = 0.519; 95% CI: 0.139–1.937; *p* = 0.329) were not significantly predicted. Emphysema was identified as a significant independent predictor of postoperative complications (χ^2^ = 5.13; *p* = 0.024). **Conclusions**: The AI-driven radiomics model demonstrated strong predictive ability for the T component and identified emphysema as a clinically relevant predictor of postoperative complications.

## 1. Introduction

Lung cancer is still a leading cause of cancer-related deaths worldwide. In 2022, lung cancer was the most frequently diagnosed cancer globally, with approximately 2.5 million new cases, accounting for 12.4% of all new cancer cases. It was also the leading cause of cancer death, with 1.8 million deaths, representing 18.7% of all cancer deaths. In Serbia, with about 7000 new cases (16.4% of all newly diagnosed cancers) and about 6000 deaths per year (24.4% of all cancer deaths), lung cancer ranks first in terms of the number of newly diagnosed cases and deaths, while globally, Serbia ranks fourth in terms of lung cancer incidence and mortality [1].

The most common histological type of lung cancer is non-small cell lung cancer (NSCLC), which accounts for about 85% of all lung cancer cases [2].

The preferred treatment for early NSCLC (stages I and II) is surgery [3]. Lobectomy has long been considered the gold standard for early NSCLC. Still, some studies have shown complementary results to sublobar resections in strictly defined cases of early NSCLC, although some meta-analyses emphasize the advantages of lobectomy in the treatment of early NSCLC [4,5,6,7].

Despite significant advances in preoperative staging, the prognosis of early NSCLC remains poor, with many patients presenting with lymph node metastases or distant metastases [8].

Lately, artificial intelligence (AI) has emerged as a valuable resource in oncology [9]. AI technology is based on extensive data processing and deep learning algorithms to independently extract information from medical images and enable precise analysis and decision-making [8]. Many studies emphasize the importance of AI in preoperative staging, the detection of nodal metastases, the prediction of postoperative outcomes, and the prediction of recurrence in early NSCLC [8,10,11,12].

The aim of this study is to investigate whether it is possible to preoperatively predict the stage of early NSCLC and the factors influencing postoperative complications using an AI-based algorithmic model.

This manuscript was prepared in accordance with the TRIPOD+AI reporting guidelines to ensure transparency, reproducibility, and clarity in AI model development and evaluation [13].

## 2. Materials and Methods

### 2.1. Patients

This retrospective study included patients with early NSCLC who underwent lobectomy by video-assisted thoracic surgery (VATS) or open surgery approach, with systematic lymph node dissection and achieving R0 resection, at the Clinic for Thoracic Surgery of the University Clinical Center of Serbia, in the period from 1 January 2019 to 31 December 2024.

Exclusion criteria from the study:

- Patients who received neoadjuvant therapy before surgery;
- Patients with early NSCLC who underwent another type of surgery;
- Patients who underwent VATS or open approach lobectomy for early NSCLC in whom the condition of R0 resection is not achieved, or do not belong to the early stage of NSCLC, i.e., in patients with a patohistological findings that corresponds to another type of primary lung malignancy that does not belong to NSCLC, metastatic disease in the lungs of another primary location or tumors of benign etiology.

### 2.2. Data Collection

We reviewed each patient’s medical records from an electronic database for preoperative clinical information, as well as postoperative information, including data from pathohistological findings of surgical materials. TNM stage was determined according to the eighth edition of the TNM stage classification for lung cancer. The collected data were compared with data extracted from CT images and analyzed using appropriate statistical methods.

### 2.3. Radiomics Extraction from CT Images

Examinations were performed on a 128-detector Somatom Definition Edge CT (Siemens Medical Systems, Erlangen, Germany). Thoracic examinations were performed according to the standard, after i.v. administration of 80–100 mL of iodine contrast, in a bolus, in the late arterial phase with reconstructions no larger than 2 mm, rotation time 0.7 s, table movement speed 38 mm/s, FOV 50 cm, 120 kV, 120–750 mAs).

**Figure 1:**
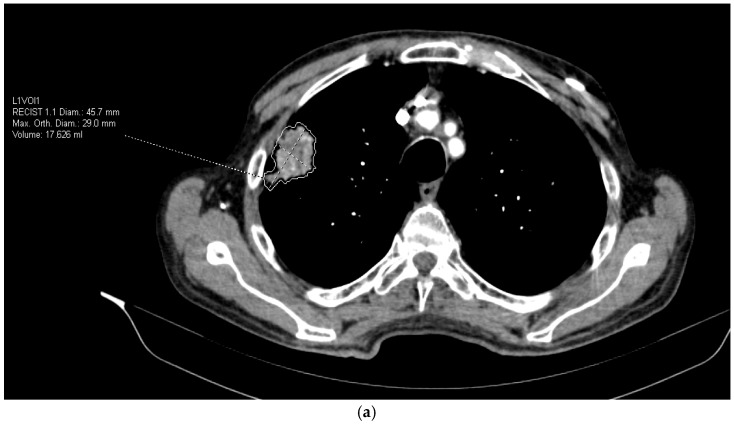

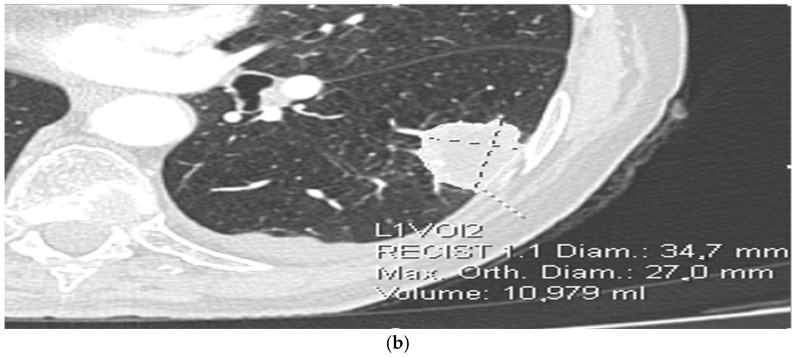
(**a**) Extraction of radiomic features from CT images. (**b**) Extraction of radiomic features from CT images.

Scans were anonymized and imported into syngo.via VB50 MM Oncology + CT Vascular AI module for automated lesion segmentation and radiomic feature extraction (Lesion Scout with Auto ID). This platform applies a deep-learning–assisted segmentation and automated feature extraction pipeline that conforms to the Image Biomarker Standardization Initiative (IBSI) principles. Quantitative parameters include first-order histogram metrics (mean, skewness, kurtosis, entropy) and texture-based features derived from gray-level co-occurrence and run-length matrices, computed on isotropic CT voxel data (1 mm^3^). All calculations used default system discretization settings and standard vendor-defined normalization, ensuring reproducible results across patients. (Figure 1a,b). Data processing were performed by the radiologists experienced in the field of thoracic radiology.

The following radiomic features were included in the model:

- Lesion morphology: maximum diameter (mm), volume (mm^3^), shape irregularity;
- Texture features: mean attenuation, entropy, skewness, kurtosis, gray-level co-occurrence metrics;
- Lung parenchyma parameters: emphysema volume fraction (<−910 HU as % of total lung volume) [14,15], presence of satellite lesions;
- Lymph node characteristics: hypervascular, round, >10 mm short axis as criteria for a pathological process;
- Lymphovascular tumor invasion characteristics: distortion of lymphatic and blood vessels or direct infiltration thereof.

All features were standardized (z-score normalization) before model training. Missing data (<5%) were imputed using median imputation.

### 2.4. Model Development

Radiomic features, combined with selected clinical predictors (age, gender, smoking, comorbidities), were used to train supervised learning models target outcomes: prediction of T component (radiologic vs. pathologic tumor diameter concordance), prediction of nodal metastases, lymphovascular invasion (LVI), and postoperative complications.

Postoperative complications were analyzed as a binary composite endpoint due to the small number of individual events.

The dataset was randomly divided into a training set (*n* = 36 cases; 70%) and a test set (*n* = 15; 30%). A regularized logistic regression model (LASSO penalty) was applied to prevent overfitting. Hyperparameters were optimized using 5-fold cross-validation on the training set, and feature selection was based on minimum cross-validated deviance. Model development was conducted using R 4.5.0 and caret, glmnet, and pROC packages (R Core Team (2024). R: A language and environment for statistical computing. R Foundation for Statistical Computing, Vienna, Austria).

### 2.5. Model Evaluation and Validation

Model performance was assessed in the independent test subset using: (1) Discrimination metrics: AUC (area under the ROC curve), accuracy, sensitivity, specificity, precision, and F1 score, (2) Calibration: Hosmer–Lemeshow goodness-of-fit test and calibration plot, (3) Explainability: feature importance ranking and SHAP (Shapley additive explanations) summary plots.

Bootstrap resampling (*n* = 1000) was used to estimate 95% confidence intervals of performance metrics. The final model was internally validated; external validation is planned in future multicenter studies.

### 2.6. Statistical Analysis

Data are presented as frequencies, mean with standard deviation, or as median with range, depending on data type and distribution. Groups were compared using parametric (*t*-test) and non-parametric tests (chi-square, Fisher’s exact test, and Mann–Whitney U test). Correlation between the variables was assessed using Spearman’s rank correlation coefficient. All *p*-values less than 0.05 were considered significant. Data were analyzed using SPSS Statistics software version 23.0 for Windows (Armonk, New York, NY, USA) and R 4.5.0 (R Core Team (2024). R: A language and environment for statistical computing. R Foundation for Statistical Computing, Vienna, Austria).

### 2.7. Ethical Aspects

Written informed consent for surgery and subsequent scientific analysis of anonymized data was obtained preoperatively from all patients. In cases where the patient had died, consent for study inclusion was obtained from the next of kin. Ethical approval was granted by the Institutional Review Board (approval No. 420/7, dated 26 December 2024), and performed in accordance with the tenets of the Declaration of Helsinki.

## 3. Results

A total of 51 patients who met the previously mentioned conditions were included in the study, of which 29 underwent open surgery and 22 VATS. In the overall cohort, 27 (52.9%) participants were male and 24 (47.1%) were female. There was no difference in gender distribution or age between patients in the open surgery and VATS groups (Table 1).

There were more smokers (*n* = 47; 94%) than nonsmokers (*n* = 3; 6%) in the overall cohort, with no significant difference in smoking status between the open surgery and VATS groups (*p* = 0.754, Table 1). The VATS group had a significantly higher forced vital capacity (FVC) compared to the open surgery group (median, 125.0 vs. 106.5, respectively), and comorbidities were more frequent in the VATS group (*p* = 0.038).

The hospital stay was significantly longer in the open surgery group compared to the VATS group (median, 7 vs. 3 days, respectively; *p* < 0.001). The number of resected lymph nodes was similar between patients undergoing open surgery and VATS (*p* = 0.667).

Complications were defined according to the European Society of Thoracic Surgeons (ESTS) guidelines, including those occurring during the hospital stay or within 30 days from the operation [16]. Postoperative complications were recorded in 10 (20%) patients, with no significant difference between the groups (*p* = 0.866).

The most common tumor localization was the right upper lobe (35% of all cases), followed by the right lower and left upper lobe (24% of each), while the least common localization was the left lower lobe with 13% and the middle lobe with 4% of all cases, with no statistically significant difference between groups (*p* > 0.05).

Data obtained by extraction from CT images are shown in Table 2. There was no difference in the presence of emphysema (*p* = 0.492), diameter (*p* = 0.588), volume of the tumor (*p* = 0.985), or in the presence of pathological lymph nodes (*p* = 0.889) and lymphovascular invasion (*p* = 0.799).

Pathohistological evaluation of the obtained material showed that the most common cancer types were adenocarcinoma (57%) and squamous cell carcinoma (35%), with large cell carcinoma present in 4%, and other rare types of NSCLC present in 4% of cases. The most common cancer stage was IB (40% of cases), followed by stage II B (22%), with stages IA and IIA present in 19% each. There was no significant difference in carcinoma type or in the tumor stage between the open surgery group and the VATS group (*p* > 0.05).

Other parameters obtained from the evaluation of pathohistological operative findings are shown in Table 3.

**Figure 2:**
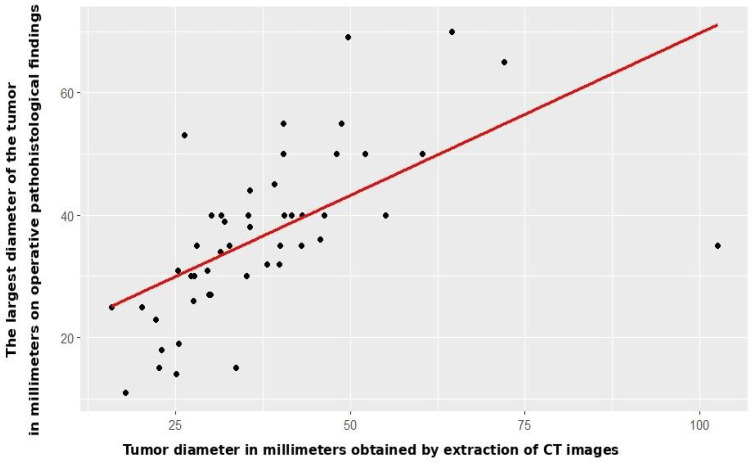
Correlation between CT and patohistological findings in relation to the T component—entire study cohort.

**Figure 3:**
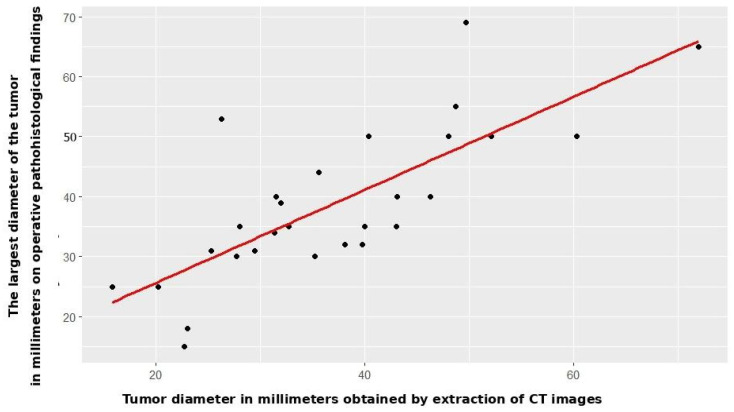
Correlation between CT and patohistological findings in relation to the T component—open surgery group.

**Figure 4:**
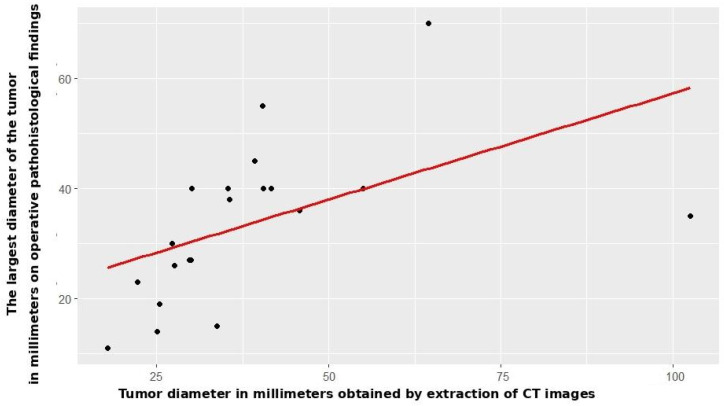
Correlation between CT and patohistological findings in relation to the T component—VATS group.

Radiomic tumor diameter strongly correlated with histopathological diameter (Spearman r_s_ = 0.766, *p* < 0.001, Figure 2), consistent across both subgroups (open: r_s_ = 0.773, *p* < 0.001; VATS: r_s_ = 0.754, *p* < 0.001, Figure 3 and Figure 4).

There was no significant difference between the open surgery group and the VATS group according to these parameters (Table 2 and Table 3).

The AI-based logistic regression model demonstrated high predictive accuracy for the T component.

**Figure 5:**
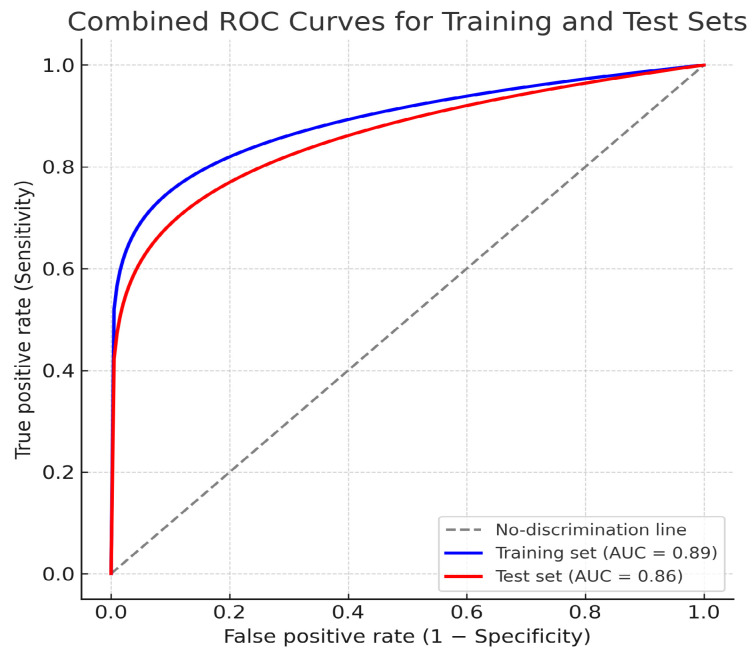
Combined receiver operating characteristic (ROC) curves for the training and test datasets for T component prediction.

Training set performance showed the following results: AUC = 0.89 (95% CI: 0.83–0.94), accuracy = 0.84, sensitivity = 0.82, specificity = 0.86, precision 0,85, and F1 score = 0.83. Test set performance showed AUC = 0.86 (95% CI: 0.78–0.93), accuracy = 0.81, sensitivity = 0.80, specificity = 0.82, precision 0.82, and F1 Score = 0.81. (Figure 5).

**Figure 6:**
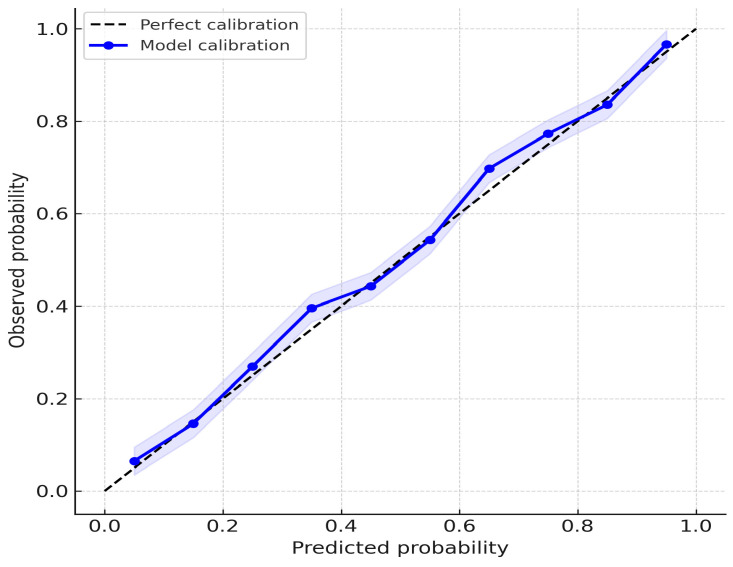
Calibration plot of the AI-based model for T-component prediction.

Calibration analysis showed no significant misfit (Hosmer–Lemeshow *p* = 0.41), and the calibration plot demonstrated good agreement between predicted and observed probabilities (Figure 6).

In relation to the N stage, univariate binary logistic regression did not single out pathological lymph nodes obtained by processing CT images as a significant predictor of pathological lymph nodes on operative pathohistological findings (OR = 0.108, 95% CI [0.011–1.069], *p* = 0.057).

In relation to these parameters, there was no statistically significant difference between the groups (Table 2 and Table 3).

**Figure 7:**
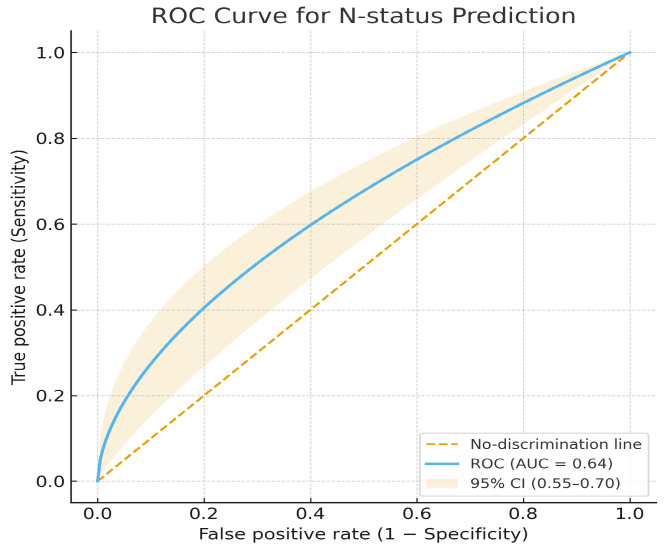
Receiver operating characteristic (ROC) curve for N-status prediction.

The model’s test-set AUC for N-stage prediction was 0.64, indicating modest discrimination. Accuracy was 0.60, sensitivity = 0.58, specificity = 0.62, precision 0.59, and F1 Score = 0.58 (Figure 7)

Radiomic detection of LVI showed no significant predictive ability relative to histopathologic results (OR = 0.519, 95% CI [0.139–1.937], *p* = 0.329).

It was also not a significant predictor in the open surgery group (OR = 0.480, 95% CI [0.076–3.029], *p* = 0.435), nor in the VATS group (OR = 0.500, 95% CI [0.060–4.153], *p* = 0.521).

**Figure 8:**
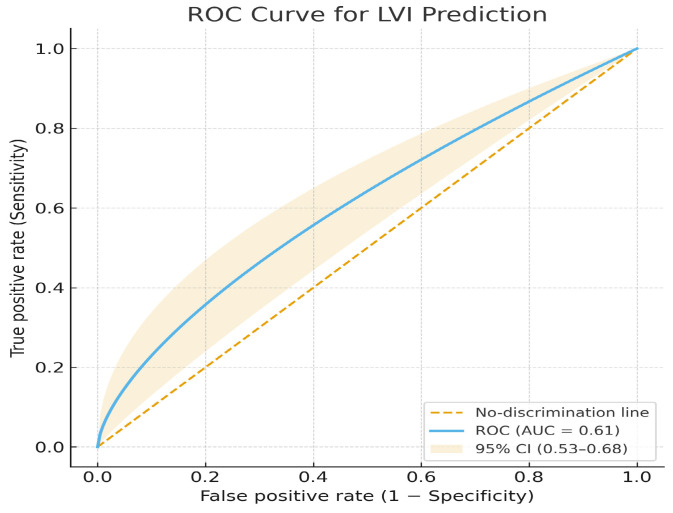
ROC curve for lymphovascular invasion (LVI) prediction.

The test-set AUC for this outcome was 0.61, showing very poor discrimination, essentially at chance level. Accuracy was 0.57, Sensitivity = 0.55, Specificity = 0.58, Precision 0.55, F1 Score = 0.55 (Figure 8).

Emphysema on preoperative CT emerged as a strong independent predictor of postoperative complications: (χ^2^ = 5.128, *p* = 0.024). Patients with emphysema had higher complication rates (notably prolonged air leak and pneumonia).

When analyzed by surgical subgroup, there was no statistical significance in any group (open surgery *p* = 0.064; VATS *p* = 0.586).

**Figure 9:**
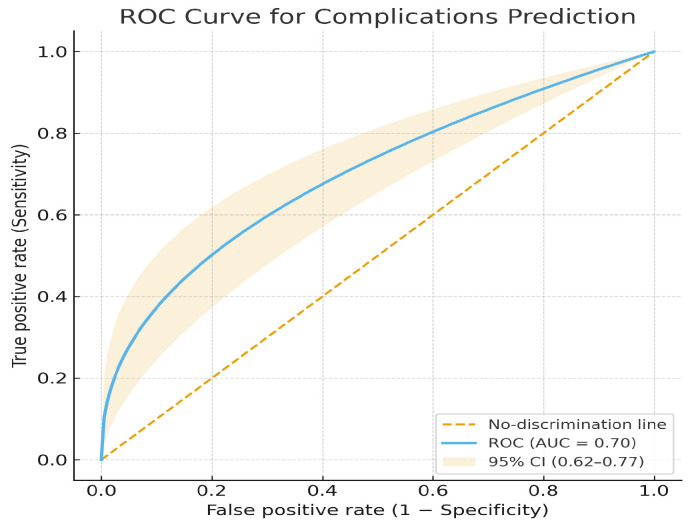
ROC curve for postoperative complications prediction.

The AI-based model demonstrated moderate discriminative performance for predicting postoperative complications (AUC = 0.70, 95% CI 0.62–0.77), with an accuracy of 0.70, sensitivity of 0.72, specificity of 0.68, precision of 0.70, and F1-score of 0.71. These findings indicate acceptable internal discrimination and a clinically meaningful predictive signal (Figure 9).

## 4. Discussion

The recurrence rate after the initial curative treatment of early NSCLC remains significant, 11.1–22%, while for stage IIIA it is 52–72% [17]; therefore, adequate initial assessment and selection of the appropriate therapeutic option are crucial.

Recently, artificial intelligence has been playing an important role in oncology, from screening and staging to predicting positive lymph nodes and even predicting outcomes [18].

Using mathematical algorithms, artificial intelligence enables early detection of disease, speeds up treatment, and significantly improves patient outcomes [19]. The application of radiomics, combined with deep learning models, has made a significant contribution to cancer staging [20].

There are several studies conducted using artificial intelligence for the staging of non-small cell lung cancer (NSCLC). Masoud et al. [10] used a convolutional neural network to classify tumor stage (T stage). An accuracy of 78% to 96% was achieved for stages T1 to T4. Aerts et al. [21] found a significant association with primary tumor stage (T stage) and overall stage in their study.

Recent studies have explored AI-based prediction across various organ systems, including MRI radiomics for post-infarction remodeling [22] and CT-radiomics combined with inflammatory markers for pulmonary nodule characterization [23].

In our study, a strong correlation was observed between the T component, as verified by pathohistological findings of the operative material, and the T component obtained by radiomics analysis of CT images. A strong correlation was verified in the whole study cohort, as well as by groups separately (open surgery/VATS).

Specifically, the strong correlation between radiomic and histopathologic tumor diameters indicates that AI-based quantitative CT analysis can approximate pathologic tumor extent preoperatively. This finding has direct relevance for surgical decision-making, particularly in borderline T lesions, where precise tumor dimensions determine both resection type and lymph node management strategy.

As emphasized in prior studies, quantitative imaging biomarkers can enhance preoperative staging consistency and reduce interobserver variability in clinical T assessment [21,24].

Pathological nodal stage is a key prognostic factor in resectable NSCLC. Metastases in mediastinal lymph nodes indicate a poor prognosis [25].

Many studies on lymph node metastases have been conducted in which lymph node size was used as a decisive criterion for predicting lymph node involvement. Decades have passed since the threshold for normal lymph nodes was proposed, and 1.0 cm is still used as the cutoff value [26].

Recently, Ziyade et al. [27] showed in a study conducted on cadavers that the diameter of a normal lymph node can be 1.0 cm for stations other than 4R and 7, but the definition of normal lymph node diameter in stations 4R and 7 can be changed to 1.5 cm and 2.0 cm.

Shimada et al. [28], in a study investigating AI-based radiomics for the prediction of lymph node metastasis in early NSCLC, showed that the 5-year recurrence-free survival rate varied from 40.2% in positive lymph nodes to 84.2% in negative lymph nodes.

Chen et al. [8], in a systematic review and meta-analysis examining the role of AI in predicting positive lymph nodes, using data from 11 studies, including 6088 patients with NSCLC, of whom 1483 had verified lymph node metastases, conclude that data capture models based on artificial intelligence algorithms have good diagnostic accuracy in predicting lymph node metastases in patients with NSCLC and could be applied in clinical practice. Other studies have shown that artificial intelligence based on multiple radiomics, deep learning, and clinical features can be effectively used to quantitatively predict presurgical N2 disease in patients with clinical stage I-II NSCLC [29].

Our work did not show a significant correlation of AI-based radiomics with the prediction of metastases in lymph nodes, both in the whole study cohort and by groups separately. One of the explanations for this result could be that CT was used for the analysis in our study, which is inferior in the diagnosis of metastases in the hilar and mediastinal lymph nodes compared to PET/CT and MRI, and thus is not in high concordance with pathological staging [30].

Lymphovascular invasion (LVI) is a negative prognostic factor in patients with early NSCLC. The presence of LVI significantly increases the risk of nodal and distant recurrence [31].

Some of the studies showed the possibility of preoperative prediction of LVI. Wang et al. [32] suggest that using a deep convolutional neural network on preoperative chest CT is a feasible approach for predicting pathological LVI in patients with NSCLC. Hosseini et al. [33] indicate that choosing an appropriate segmentation method and machine learning algorithm can be useful in successfully predicting LVI in NSCLC patients with high accuracy using PET radiomic analysis.

Our examination did not show a statistically significant correlation between lymphovascular invasion, as verified by radiomics extraction of CT images, and lymphovascular invasion on pathohistological operative findings, in the whole cohort and in separate groups. One of the reasons for this result may be that only CT was used in the analysis. Both CT and PET/CT play a role in predicting LVI in NSCLC, but PET/CT offers superior accuracy. PET/CT, particularly when combined with radiomics, demonstrates better predictive power than CT alone for LVI, potentially influencing treatment decisions [34].

The significance of the appearance of emphysema on preoperative chest CT is highlighted in numerous studies as a significant factor affecting the appearance of postoperative complications, primarily prolonged air leakage. Murakami et al. [14], in a study conducted on 284 patients who underwent lobectomy for lung cancer, concluded that the grade of emphysema on preoperative CT is the best predictor of postoperative prolonged air leakage, which negatively affects early postoperative outcomes. In a retrospective study by Sato et al. [15], at 566 consecutively operated early-stage lung cancer patients, noticed that the presence of emphysema affects long-term outcomes and the development of postoperative complications in early-stage lung cancer patients. Kitazawa et al. [35] emphasize the importance of the presence of a low-attenuation area (LAA) on CT, which correlates well with the presence of pulmonary emphysema, concluding that LAA can be used as a predictor of postoperative respiratory complications in patients previously operated on for lung cancer.

Our results are complementary to published works on this topic, highlighting emphysema as a significant factor in the occurrence of postoperative complications. Although prolonged air leak and pneumonia were the most frequent complications, their low incidence did not permit reliable subgroup statistical analysis. The absence of a statistically significant correlation between emphysema and postoperative complications, when analyzed separately by group (open surgery/VATS), can be explained by the small number of frequencies.

There are several limitations that should be pointed out in this study. First, it was a retrospective study from a single institution, with a limited number of patients. Nevertheless, internal validation using regularized regression and cross-validation confirmed stable model behavior and consistent effect estimates, indicating methodological soundness despite the small cohort size. No external comparator (such as conventional clinical or imaging staging) was included in this study; therefore, the present analysis does not allow direct benchmarking of AI performance against traditional methods. Second, not all preoperative CT scans of patients were successfully processed using our radiomic analysis. Because a small number of CT scans could not be processed, a minimal amount of data loss occurred. However, the proportion of missing data was below 5%, and imputation was performed using robust statistical techniques, minimizing bias and preserving internal validity. Future studies with larger sample sizes and multi-center data will help verify the robustness of the model against incomplete imaging data. Third, our study used only CT imaging. PET/CT is very useful for predicting prognosis and detecting pathological invasive factors, including lymph node metastases, with higher specificity than CT imaging.

## 5. Conclusions

The results of this study suggest that the AI-based algorithmic model can be an independent factor for predicting the T component in the staging of early non-small cell lung cancer, as well as lung emphysema as a predictive factor for the occurrence of postoperative complications. Considering the limited number of patients in this study, further research is necessary.

## Figures and Tables

**Table 1:** Demographic and clinical characteristics of patients in the open surgery and VATS groups.

Patient Characteristics	Open Surgery	VATS	*p*
**Gender**			
Male	15 (51.7%)	12 (54.5%)	0.842
Female	14 (48.3%)	10 (45.5%)	
Age *	70 (56–82)	70 (36–80)	0.871
**Smoking**			
Nonsmoker	2 (6.9%)	1 (4.8%)	0.754
Smoker	27 (93.1%)	20 (95.2%)	
**Lung function**			
FEV1 (%) *	92.5 (59.0–139.0)	96.0 (79.0–155.0)	0.070
FEV1 (liters) *	2.32 (1.32–3.12)	2.45 (1.78–4.69)	0.123
FVC (%) *	106.5 (89.0–145.0)	125.0 (90.0–389.0)	0.009
TIFF (%) *	68.79 ± 10.06	68.06 ± 10.66	0.812
DLCO (%) **	67.5 ± 17.6	77.6 ± 23.3	0.075
KCO (%) **	71.2 ± 20.3	77.9 ± 24.2	0.389
**Comorbidities** *	1 (1–5)	3 (1–5)	0.038
Length of hospitalization (days) *	7 (4–33)	3 (2–8)	<0.001
Number of resected lymph nodes *	9 (2–23)	7.5 (2–32)	0.667
**Postoperative complications, yes**	6 (20.7%)	4 (19.1%)	0.866
**Air leak, yes**	2 (6.9%)	0 (0%)	0.503
**Empyema, yes**	1 (100%)	0	1.000
**Pneumonia, yes**	1 (100%)	0	1.000
**Wound dehiscence, yes**	1 (50%)	1 (50%)	1.000

* median (range); ** mean ± standard deviation; FEV1—Forced expiratory volume in 1 s; FVC—Forced vital capacity.

**Table 2:** Patients’ characteristics according to CT findings.

Patient Characteristics	Overall Cohort	Open Surgery	VATS	*p*
**Emphysema**				
No	25 (49%)	13 (44.8%)	12 (54.5%)	0.492
Yes	26 (51%)	16 (55.2%)	10 (45.4%)	
Diameter of the tumor *	35.4 (15.8–102.6)	35.6 (15.8–72.0)	34.6 (17.8–102.6)	0.588
Volume of the tumor *	11.468 (1.565–217.276)	11.588 (1.565–158.455)	11.935 (1.851–217.276)	0.985
**Pathological lymph nodes**				
No	33 (64.7%)	19 (65.5%)	14 (63.6%)	0.889
Yes	18 (35.3%)	10 (34.5%)	8 (36.4%)	
**Lymphovascular invasion**				
**No**	38 (74.5%)	22 (75.8%)	16 (72.7%)	0.799
Yes	13 (25.5%)	7 (24.2%)	6 (27.3%)	
**Satellite lesion (same lobe)**				
No	41 (80.4%)	24 (82.8%)	17 (77.3%)	0.625
Yes	10 (19.6%)	5 (17.2%)	5 (22.7%)	
**Satellite lesion, other lobe**				
No	49 (96%)	28 (96.6%)	21 (95.5%)	1.000
Yes	2 (4%)	1 (3.4%)	1 (4.5%)	

* median (range).

**Table 3:** Data obtained from the evaluation of operative pathohistological findings.

Patient Characteristics	Overall Cohort	Open Surgery	VATS	*p*
**Pathological lymph nodes**				
No	46 (90.2%)	25 (86.2%)	21 (95.5%)	0.271
Yes	5 (9.8%)	4 (13.8%)	1 (4.5%)	
Tumor size, largest diameter (mm) *	36.7 ± 13.5	38.9 ± 12.8	35.6 ± 14.3	0.184
**Lymphovascular invasion**				
No	27 (54%)	12 (41.4%)	15 (71.4%)	0.035
Yes	23 (46%)	17 (58.6%)	6 (28.6%)	

* mean ± standard deviation.

## Data Availability

The data that support the findings of this study are available from the first author (Z.G.) upon reasonable request.
